# Identification of circulating microRNA biomarkers in pulmonary hypertension of group 2 for early detection

**DOI:** 10.3389/fcvm.2026.1704427

**Published:** 2026-02-20

**Authors:** Xiaoyao Qin, Liqin Zheng, Huan Zhao, Qiaozhi Li, Liming Wang, Qianqian Lu, Peng Chen, Zhifeng Xue, Zhengwei Dong

**Affiliations:** 1 Henan University of Chinese Medicine, Zhengzhou, China; 2 Heart Center/National Regional (Traditional Chinese Medicine) Cardiovascular Diagnosis and Treatment Center, The First Affiliated Hospital of Henan University of CM, Zhengzhou, China; 3 Department of Respiratory and Critical Care Medicine, Shanxi Province Fenyang Hospital, Fenyang, Shanxi, China; 4 Tianjin University of Traditional Chinese Medicine, Tianjin, China; 5 Shanxi Academy of Medical Sciences, Third Hospital of Shanxi Medical University, Tongji Shanxi Hospital, Shanxi Bethune Hospital, Taiyuan, China

**Keywords:** biomarkers, early detection, microRNA, pulmonary hypertension, pulmonary hypertension of group 2

## Abstract

**Background:**

Pulmonary hypertension (PH) is a progressive cardiopulmonary disorder with high morbidity and mortality, yet non-invasive diagnostic biomarkers remain scarce. Circulating microRNAs (miRNAs) have emerged as promising biomarkers for various diseases, but their role in Group 2 PH (due to left heart disease) is poorly understood.

**Methods:**

We performed high-throughput miRNA sequencing on plasma samples from 10 healthy controls (C group), 10 patients with coronary heart disease (CHD group), and 10 patients with coronary heart disease complicated by PH (PH group). Differential miRNA expression was analyzed, and candidate miRNAs were validated by RT-qPCR. Correlations with pulmonary artery pressure parameters (Tricuspid regurgitation maximum velocity (TRVmax) and systolic pulmonary artery pressure (SPAP) were assessed, and bioinformatic analyses were conducted to predict target genes and functional pathways.

**Results:**

We identified 575 differentially expressed miRNAs across the three groups. Eleven miRNAs were significantly dysregulated in PH patients compared to CHD and C groups. Among these, five miRNAs (mmu-miR-452-3p_1ss20GA, hsa-miR-10a-3p_R-1, hsa-miR-21-5p, hsa-miR-1287-5p_R + 1, and bta-mir-1246-p5_1ss18AG) showed strong positive correlations with TRVmax and SPAP. Receiver operating characteristic (ROC) curve analyses revealed high diagnostic accuracy. Functional enrichment analysis indicated involvement in key signaling pathways such as p53, PPAR, TGF-β, JAK-STAT, and MAPK.

**Conclusion:**

Our study identifies a panel of circulating miRNAs as potential non-invasive biomarkers for diagnosing Group 2 PH and provides insights into their roles in PH pathogenesis, offering new avenues for therapeutic intervention.

## 1 Background

Pulmonary hypertension (PH) is a cardiopulmonary disorder characterized by a progressive increase in pulmonary arterial pressure, ultimately leading to right heart failure. Left heart-related PH constitutes the second major category of PH (1). It affects at least 1% of the global population, with middle- and low-income countries likely bearing a disproportionately greater burden (2). The group 2 PH accounts for approximately half of all cases of PH (3). The pathological mechanisms underlying PH have not yet been fully elucidated. Currently approved drugs for PH have not demonstrated a significant improvement in patient survival rates (4). Right heart catheterization remains the gold standard for diagnosing PH and classifying its subtypes, but it is costly and has limited applicability. Echocardiography serves as a key non-invasive screening tool and is widely used in clinical practice. Additional auxiliary diagnostic methods include chest x-ray, cardiopulmonary exercise testing, and MRI (5). There remains a lack of specific, efficient, and cost-effective physicochemical detection methods for PH.

MicroRNAs (miRNAs) are endogenous non-coding single-stranded RNA molecules approximately 22 nucleotides in length that play indispensable roles in post-transcriptional gene regulation (6). They have garnered significant attention as potential novel diagnostic tools and even therapeutic agents. miRNAs are involved in modulating the progression of PH (7), and dysregulation of miRNA function can enhance the proliferation of endothelial cells, smooth muscle cells, and adventitial cells in the pulmonary arteries, thereby promoting the development of PH (8). Research on miRNAs in lung tissues of patients with pulmonary arterial hypertension (PAH) is relatively comprehensive. Specific miRNAs, including miR-150, miR-204, and miR-124, have been identified as predictors of survival and indicators of PAH severity (9–11). However, studies on circulating miRNAs in group 2 PH patients remain insufficient.

In this study, we performed high-throughput omics profiling and experimental validation of circulating miRNAs in plasma from patients with PH due to left heart disease. This study was designed to identify reliable circulating miRNA markers for this PH subtype, with the ultimate goal of providing a convenient and economical diagnostic method for group 2 PH and proposing novel therapeutic strategies.

## 2 Methods

### 2.1 Patient selection and study population

This study was approved by the Ethics Committees of Guang'anmen Hospital, China Academy of Chinese Medical Sciences (registration No. 2022-106-KY), and written informed consent was obtained from all participants prior to sample collection and data acquisition.

Patient recruitment was conducted between July 2021 and September 2021. A total of 48 patients with coronary heart disease (CHD) were initially screened for eligibility. From this pool, participants were sequentially enrolled and assigned to the study groups based on the following workflow: PH was diagnosed according to the American Thoracic Society/European Respiratory Society and European Society of Cardiology consensus criteria. Echocardiography identified patients at moderate to high risk for PH, defined by a tricuspid regurgitation peak velocity (TRVmax) > 280 cm/s or a systolic pulmonary artery pressure (SPAP) > 36 mmHg (12). All echocardiographic assessments were performed by the same experienced cardiologist, and SPAP was calculated from the maximum tricuspid regurgitation velocity.

Following the application of all clinical and echocardiographic inclusion/exclusion criteria, a final cohort of 10 CHD patients meeting the PH criteria (PH group) and 10 CHD patients without PH (CHD group) was consecutively selected. At the same time, 10 healthy individuals who underwent physical examinations at the examination center were recruited for our study as controls, excluding the patients with acute phase diseases, serious primary diseases of the liver, kidney, hematopoietic system, acute infections, and pregnant or lactating women. Plasma samples have been collected from all candidates.

### 2.2 miRNA-sequencing analysis

miRNA was extracted from plasma using MolPure® Serum/Plasma miRNA Kit (YEASEN, 19332ES50) following the manufacturers' instructions. sRNA sequencing library preparation was performed using the TruSeq Small RNA Sample Prep Kits (Illumina, San Diego, USA). After library preparation, the constructed libraries were sequenced using the Illumina HiSeq 2,000/2,500. miRNA data analysis was performed using ACGT101-miR (Hangzhou Lianchuan Biotechnology Co., Ltd, v4.2). The 3′ adapters and junk sequences were removed to obtain clean data. The sRNA length was retained within the range of 18–26 nt, and the remaining sequences were aligned with mRNA, RFam, and Repbase databases (excluding miRNA) and filtered. The reads obtained after length filtering and RFam database filtering were aligned with the precursor and genome for miRNA identification. To identify a broader spectrum of potential biomarkers, the small RNA sequences obtained from sequencing were aligned against the precursor and mature miRNA sequences from multiple species contained in the miRBase database (v22.1). miRNAs aligning to non-human species were considered highly conserved homologs of the corresponding human miRNAs or novel human miRNA candidates. Fold Change > 1.5 and *P*-value < 0.05 were considered to be significant differences.

### 2.3 Prediction of miRNA target genes

The prediction of miRNA target genes was carried out by OmicStudio tool at https://www.omicstudio.cn/tool based on TargetScan (8.0) and Miranda (3.3a), and the screening threshold was TargetScan_score  ≥ 50 and miranda_Energy < −10. [GSTAr (1.0), MFEratio > 0.65 and AllenScore < 4; PsRobot (1.2), Score < 2.5].

### 2.4 Real-time quantitative PCR (RT-qPCR) analysis

After miRNA was quantified and reverse transcribed using the tailing method (Sangon Biotech, B532451), quantitative RT-PCR was performed with SGExcel Universal SYBR qPCR Mix (Sangon Biotech, B532958) in the Real-time fluorescent quantitative PCR instrument (Roche, LightCycler 480). All gene expression data were presented as a relative expression calculated by the 2^−ΔΔCT^ method. The primer sequences for target genes are listed in Table 1.

**Table 1:** Primer sequences for RT-qPCR.

Primer name	Forward primer (5′-3′)
hsa-miR-6734-5p-FORWARD	TTGAGGGGAGAATGAGGTGGAGA
hsa-miR-18b-5p_R-2-FORWARD	GCTAAGGTGCATCTAGTGCAGTT
hsa-miR-1468-5p_R + 1-FORWARD	CTCCGTTTGCCTGTTTCGCTGA
hsa-miR-1270-FORWARD	CTGGAGATATGGAAGAGCTGTGT
bta-miR-11980_L-1R-1_1ss4CG-FORWARD	GAACGGGCTTGGCGGA
PC-5p-148323_25-FORWARD	CGTTCAGTTGCTGGCGTAGAC
mmu-miR-452-3p_1ss20GA-FORWARD	CGCTCAGTCTCATCTGCAAAGAAGT
hsa-miR-874-3p-FORWARD	CCCTGGCCCGAGGGAC
hsa-miR-3611_R-2-FORWARD	CGCCGTTGTGAAGAAAGAAATTCT
hsa-miR-21-5p-FORWARD	CCGTAGCTTATCAGACTGATGTTGA
hsa-miR-145-5p_R-2-FORWARD	CGTCCAGTTTTCCCAGGAATCC
hsa-miR-145-3p_L-2R + 1-FORWARD	CGCGATTCCTGGAAATACTGTTCTT
hsa-miR-1287-5p_R + 1-FORWARD	TGCTGGATCAGTGGTTCGAGTCT
hsa-miR-497-5p-FORWARD	CAGCAGCACACTGTGGTTTGT
hsa-miR-30a-5p_R-1-FORWARD	GCTGTAAACATCCTCGACTGGAA
hsa-miR-1246_L-2R + 1-FORWARD	GGCTGGATTTTTGGAGCAGGG
hsa-miR-10a-3p_R-1-FORWARD	GCGCAAATTCGTATCTAGGGGAAT
bta-mir-1246-p5_1ss18AG-FORWARD	CGTGGATTTTTGGAGCAGGGAG
U6-FORWARD	AGAGAAGATTAGCATGGCCCCTG

### 2.5 Biological process and pathway analysis

Heatmaps were plotted by https://www.bioinformatics.com.cn (last accessed on 10 Dec 2024), an online platform for data analysis and visualization. The *P* value < 0.05 was considered statistically significant.

### 2.6 Protein-protein interaction (PPI) analysis

The PPI analysis of differentially expressed genes was based on the STRING database (Version: 12.0).

### 2.7 Receiver operating characteristic (ROC) curve analysis

ROC curves was performed using the OmicStudio tools at https://www.omicstudio.cn/tool/58. The results were expressed as area under the curve (AUC) with 95% confidence interval, and sensitivity and specificity. Data were considered to be statistically significantly different if *P* < 0.05.

### 2.8 Statistical analysis

Statistical analysis was performed using SPSS 26.0 software. Continuous data were assessed for normality and homogeneity of variance, with normally distributed data expressed as mean ± SEM and non-normally distributed data as median. For multiple-group comparisons, one-way ANOVA (with Tukey's *post hoc* test) was used if assumptions were met; otherwise, the Kruskal–Wallis test (with Dunn's *post hoc* test) was applied. For two-group comparisons, independent *t*-tests were used for normally distributed data, while the Mann–Whitney *U* test was employed for non-parametric data. A *P*-value < 0.05 was considered statistically significant.

## 3 Results

### 3.1 Circulating miRNA transcriptomics analysis in patients with group 2 PH

**Figure 1:**
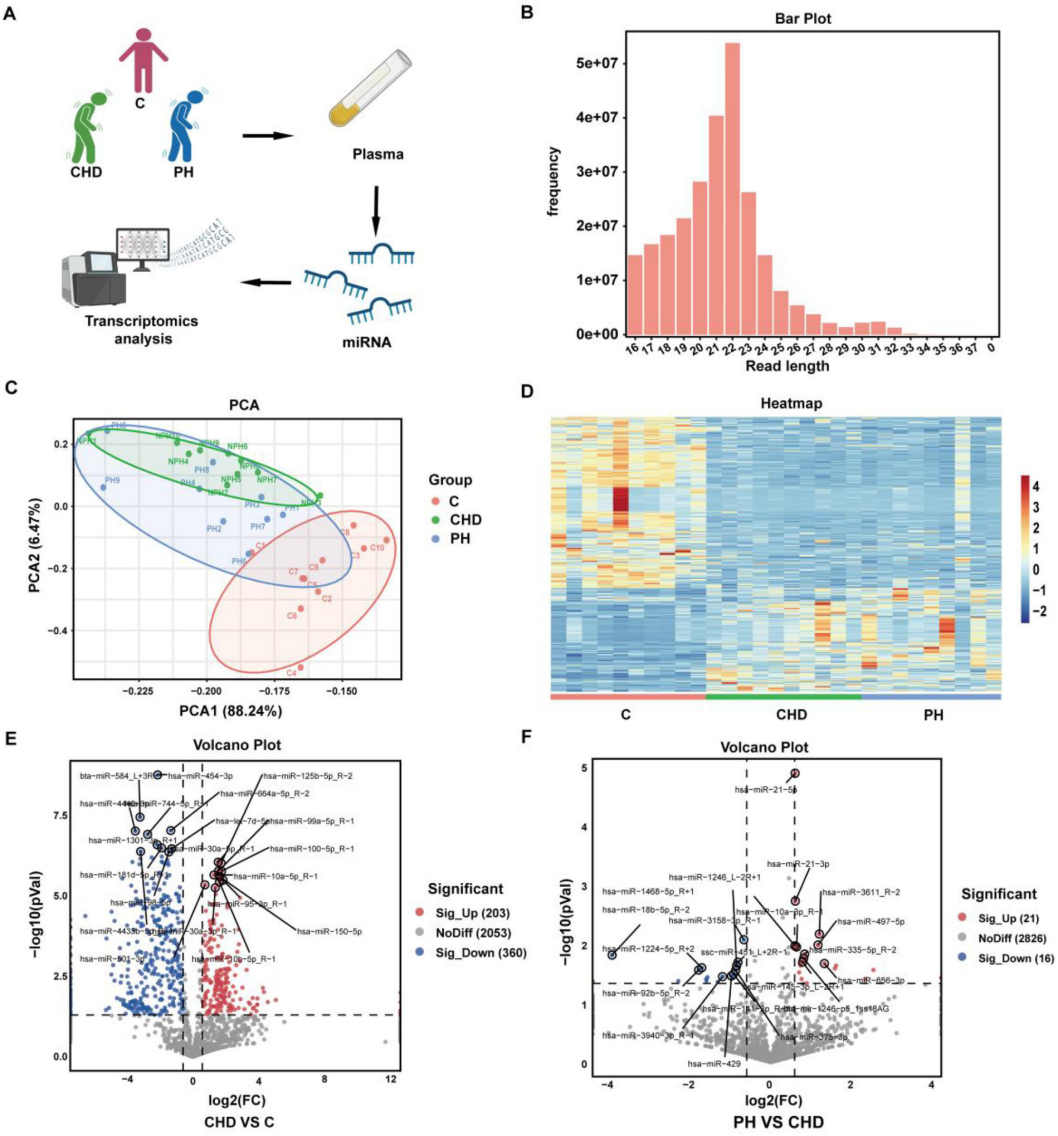
Circulating miRNA transcriptomics analysis in patients with group 2 PH. **(A)** Protocol for transcriptomics analysis. **(B)** miRNA length statistics. **(C)** Principal component analysis diagram of the C, CHD, and PH groups. **(D)** Heat map showing differentially expressed circulating miRNAs in the C, CHD, and PH groups. *n* = 9–10, Fold Change ≥ 1.5. *P* < 0.05 was considered statistically significant. **(E)** Volcano map of circulating miRNAs between CHD and C groups. **(F)** Volcano map of circulating miRNAs between PH and CHD.

Plasma was isolated from 10 healthy individuals, 10 patients with CHD, and 10 patients with CHD with PH. The baseline information for the three groups of patients is presented in Table 1. miRNAs were extracted and subjected to transcriptome sequencing (Figure 1A). There was no significant difference in age and ejection fraction between the three groups (Table 2). The lengths of the identified miRNAs were counted, and the lengths were concentrated at 18–22 nt (Figure 1B). Principal Component Analysis (PCA) was performed by miRNA expression information of the samples, R = 0.6827 (Figure 1C). A total of 575 differential miRNAs were present in the comparison between the three groups (Figure 1D). 563 differential miRNAs were present in the comparison between CHD and C group, of which 203 were up-regulated and 360 were down-regulated (Figure 1E). 37 differential miRNAs were present in the comparison between PH and CHD groups, of which 21 were up-regulated and 16 were down-regulated (Figure 1F).

**Table 2:** Baseline characteristics of the study participants.

Group	Age	Sex	TRVmax (cm/s)	SPAP (mmHg)	EF (%)	Past History
C1	67	Man	-	-	61	
C2	63	Man	-	-	62	
C3	62	Man	-	-	70	
C4	59	Man	-	-	67	
C5	59	Man	-	-	61	
C6	59	Man	-	-	64	
C7	58	Man	-	-	71	
C8	57	Man	-	-	67	
C9	57	Man	-	-	69	
C10	56	Man	-	-	60	
CHD1	72	Man	-	-	62	CHD, Hypertension, T2DM,
CHD2	67	Man	-	-	64	CHD, Hypertension, T2DM, Hyperlipidemia
CHD3	58	Man	-	-	58	CHD, Hypertension, Hyperlipidemia,
CHD4	67	Man	208	22	59	CHD, Hypertension, T2DM, Hyperlipidemia
CHD5	71	Man	-	-	63	CHD, Hypertension, T2DM, Hyperlipidemia
CHD6	55	Man	-	-	61	CHD, Hypertension, T2DM, Hyperlipidemia
CHD7	64	Man	-	-	60	CHD
CHD8	71	Man	-	-	58	CHD, Hypertension, T2DM, Hyperlipidemia
CHD9	72	Man	-	-	60	CHD, Hypertension, Hyperlipidemia
CHD10	64	Man	200	21	62	CHD, Hypertension, Hyperlipidemia
PH1	72	Man	370	65	57	CHD, Hypertension, Hyperlipidemia
PH2	69	Man	376	61	35	CHD, Hypertension, T2DM, Hyperlipidemia
PH3	74	Man	300	51	58	CHD, Hypertension, T2DM, Hyperlipidemia
PH4	67	Man	362	58	46	CHD, T2DM, Hyperlipidemia
PH5	72	Man	298	45	40	CHD, Hypertension, T2DM
PH6	40	Man	334	55	48	CHD, T2DM, Hyperlipidemia
PH7	74	Man	365	63	50	CHD, Hyperlipidemia
PH8	68	Man	233	36	44	CHD, Hypertension, T2DM
PH9	66	Man	353	60	57	CHD, Hypertension, T2DM, Hyperlipidemia
PH10	56	Man	277	35	62	CHD, Hypertension

For patients presenting with normal pulmonary arteries, TRVmax and SPAP values were not assessed or recorded.

### 3.2 Differential expression analysis of circulating miRNAs in group 2 PH

**Figure 2:**
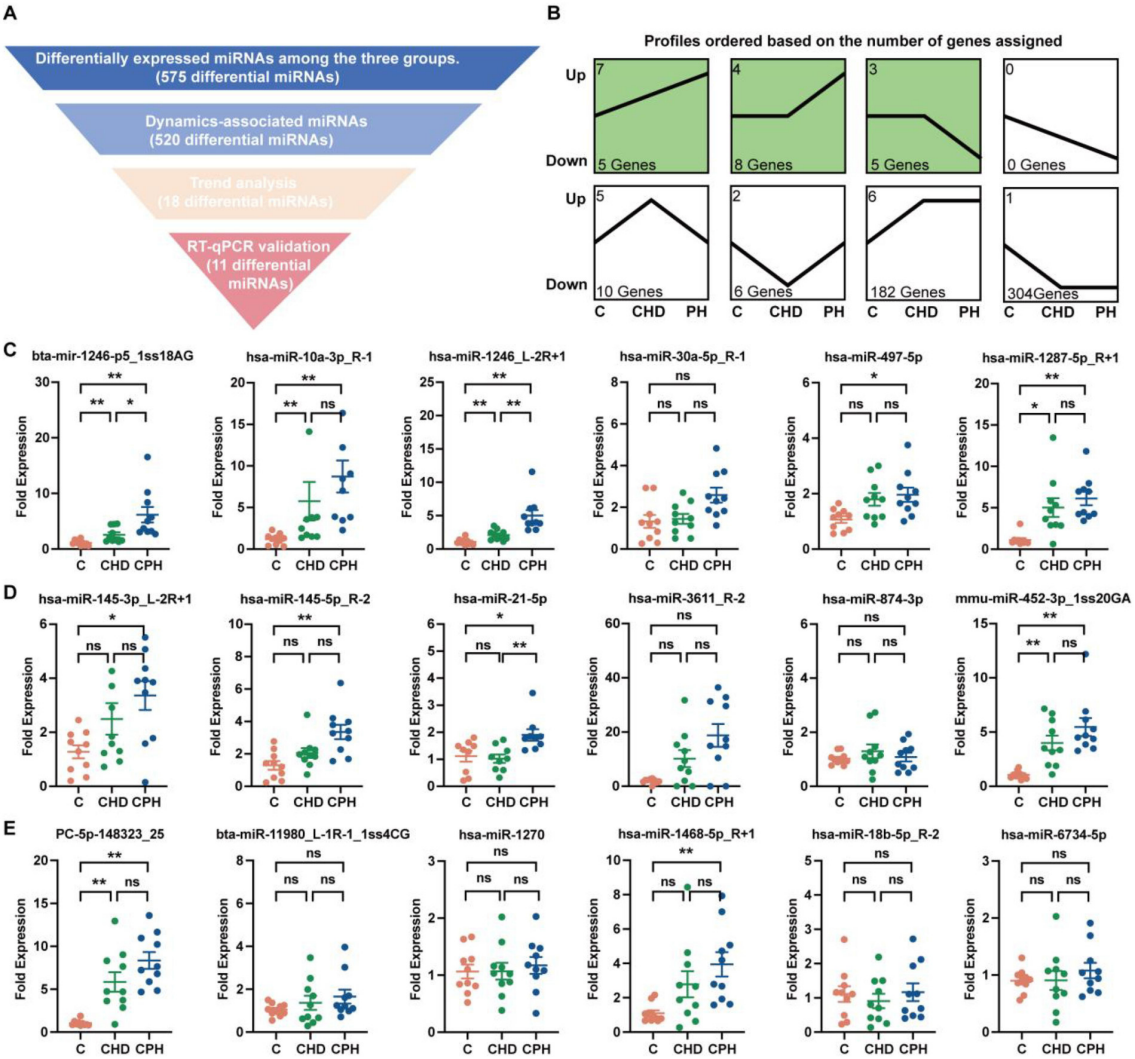
Differential expression analysis of circulating miRNAs in group 2 PH. **(A)** Differentially expressed miRNA screening strategy diagram. **(B)** Trend analysis of differential circulating miRNAs between the three groups. **(C–E)** RT-qPCR validation of differentially expressed miRNAs in clusters 7, 4, and 3 of trend analysis (*n* = 9–10). *, *P* < 0.05, **, *P* < 0.01.

Comparative analysis across the three groups identified 575 differentially expressed miRNAs. Subsequently, 520 miRNAs were selected based on their dynamic expression patterns aligned with the progression from C to CHD and then to PH. Further refinement using transcriptomic data yielded 18 candidate miRNAs exhibiting consistent trends across disease stages (Figures 2A,B). RT-qPCR validation of the 18 candidate miRNAs confirmed that 11 of them exhibited statistically significant differential expression across the three groups. The validated miRNAs include bta-mir-1246-p5_1ss18AG, hsa-miR-10a-3p_R-1, hsa-miR-1246_L-2R + 1, hsa-miR-497-5p, hsa-miR-1287-5p_R + 1, hsa-miR-145-3p_L-2R + 1, hsa-miR-145-5p_R-2, hsa-miR-21-5p, mmu-miR-452-3p_1ss20GA, pc-5p-148323_25, and hsa-miR-1468-5p_R + 1 (Figures 2C–E).

### 3.3 Analysis of the correlation between differential expression analysis of circulating miRNAs and pulmonary artery pressure in group 2 PH

**Figure 3:**
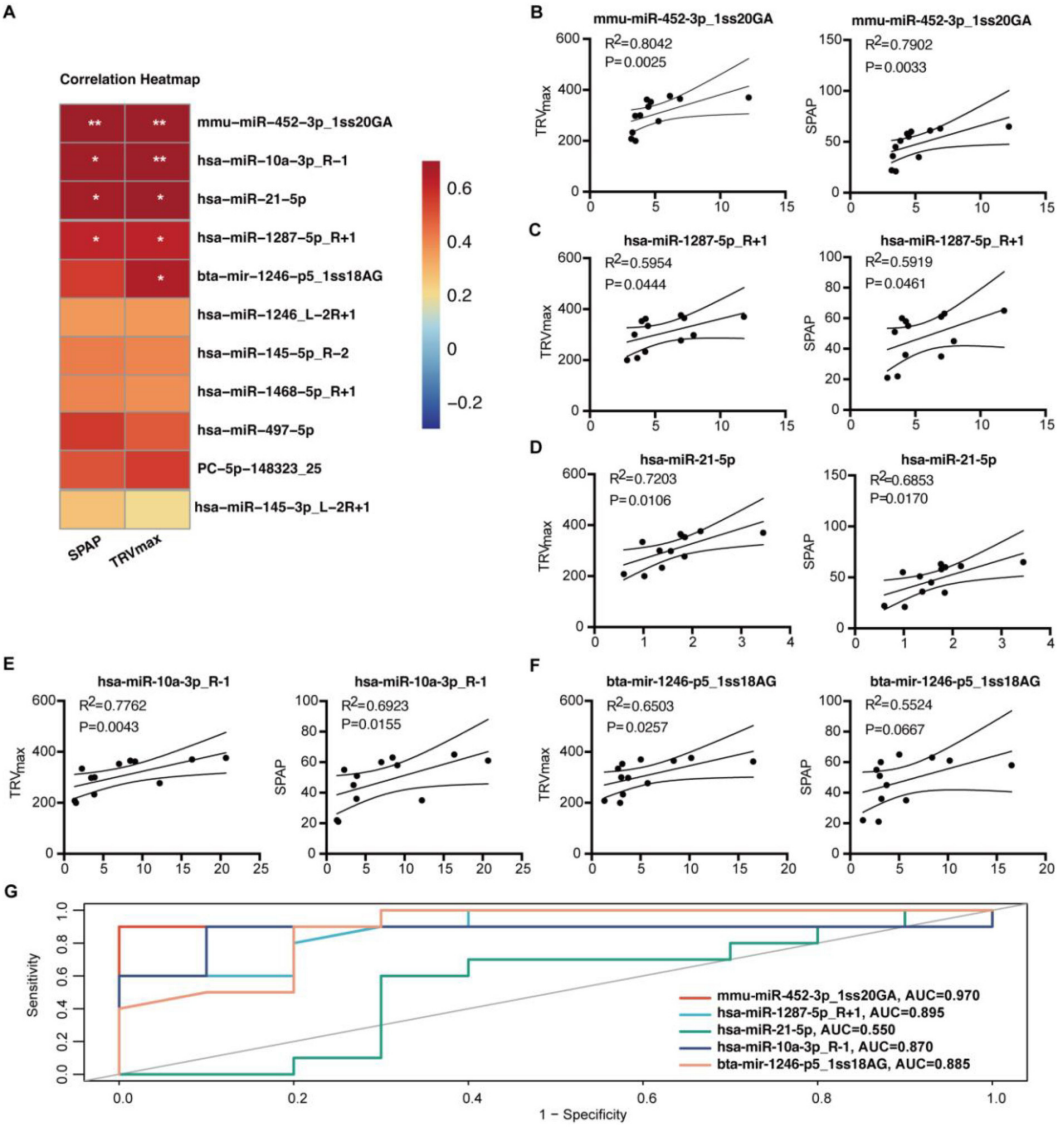
Analysis of the correlation between differential expression analysis of circulating miRNAs and pulmonary artery pressure in group 2 PH. **(A)** Heat map showing the correlation between the expression levels of 18 differentially circulating miRNAs and SPAP and TRVmax. **(B–F)** Correlation analysis between the expression levels of differentially circulating miRNAs and SPAP and TRVmax (Top 5). **(G)** ROC curve analysis of 5 most relevant differentially circulating miRNAs for diagnosing PH.

Among the 18 candidates differentially expressed miRNAs, 11 circulating miRNAs validated by RT-qPCR were correlated with TRVmax and SPAP. The results showed that the expression of mmu-miR-452-3p_1ss20GA, hsa-miR-10a-3p_R-1, hsa-miR-21-5p, hsa-miR-1287-5p_R + 1, and bta-mir-1246-p5_1ss18AG were positively correlated with TRVmax values, and the expression of mmu-miR-452-3p_1ss20GA, hsa-miR-10a-3p_R-1, hsa-miR-21-5p, and hsa-miR-1287-5p_R + 1 expression were positively correlated with SPAP values (Figures 3A–F). ROC curve analyses of the five most relevant differential circulating miRNAs for the diagnosis of PH. ROC curve analyses revealed promising diagnostic accuracy for several miRNAs: the AUC for mmu-miR-452-3p_1ss20GA was 0.970, for hsa-miR-1287-5p_R + 1 was 0.895, for hsa-miR-10a-3p_R-1 was 0.87, and for bta-mir-1246-p5_1ss18AG was 0.885. In contrast, hsa-miR-21-5p showed limited discriminatory capacity (AUC = 0.55) (Figure 3G). The miRNAs exhibited a spectrum of diagnostic accuracies. The mmu-miR-452-3p_1ss20GA showed outstanding discrimination, while hsa-miR-1287-5p_R + 1, bta-mir-1246-p5_1ss18AG, and hsa-miR-10a-3p_R-1 displayed good to moderate performance. In contrast, hsa-miR-21-5p showed limited discriminatory capacity.

### 3.4 Biological effects of target proteins of circulating miRNA biomarkers in group 2 PH

**Figure 4:**
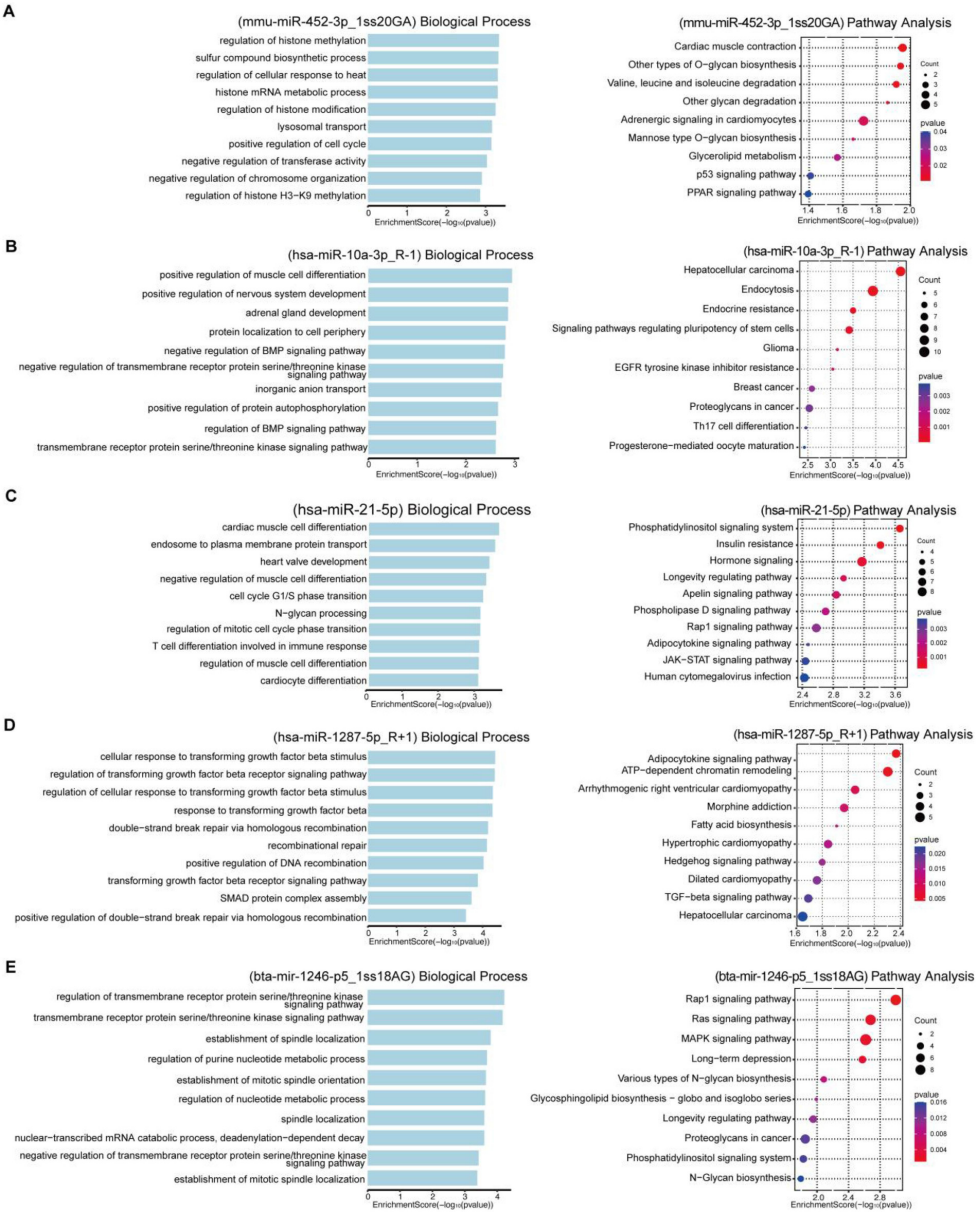
Biological effects of target proteins of circulating miRNA biomarkers in group 2 PH. **(A–E)** Biological process and pathway analysis of target proteins of mmu-miR-452-3p_1ss20GA, hsa-miR-10a-3p_R-1, hsa-miR-21-5p, hsa-miR-1287-5p_R + 1 and bta-mir-1246-p5_1ss18AG.

The functions of five circulating miRNA biomarkers were investigated. To predict the genes targeted by most abundant miRNAs, two computational target prediction algorithms (TargetScan score ≥ 50 and miranda Energy < −10) were used to identify miRNA binding sites. Finally, the data predicted by both algorithms were combined and the overlaps were calculated. Target proteins were subjected to Biological Process Analysis and Pathway Analysis. Functional analysis revealed that mmu-miR-452-3p_1ss20GA was enriched in biological processes related to histone methylation and was implicated in the regulation of the p53 and PPAR signaling pathways (Figure 4A). The hsa-miR-10a-3p_R-1 has a role in regulating muscle cell differentiation (Figure 4B). The hsa-miR-21-5p has a role in regulating muscle cell differentiation and JAK-STAT signaling pathways (Figure 4C). The hsa-miR-1287-5p_R + 1 has a role in regulating TGF-beta signaling pathway (Figure 4D). The bta-mir-1246-p5_1ss18AG has a role in regulating Rap1 signaling pathway, Ras signaling pathway and MAPK signaling pathway (Figure 4E).

### 3.5 Interactions of target proteins of circulating miRNA biomarkers in patients with group 2 PH

**Figure 5:**
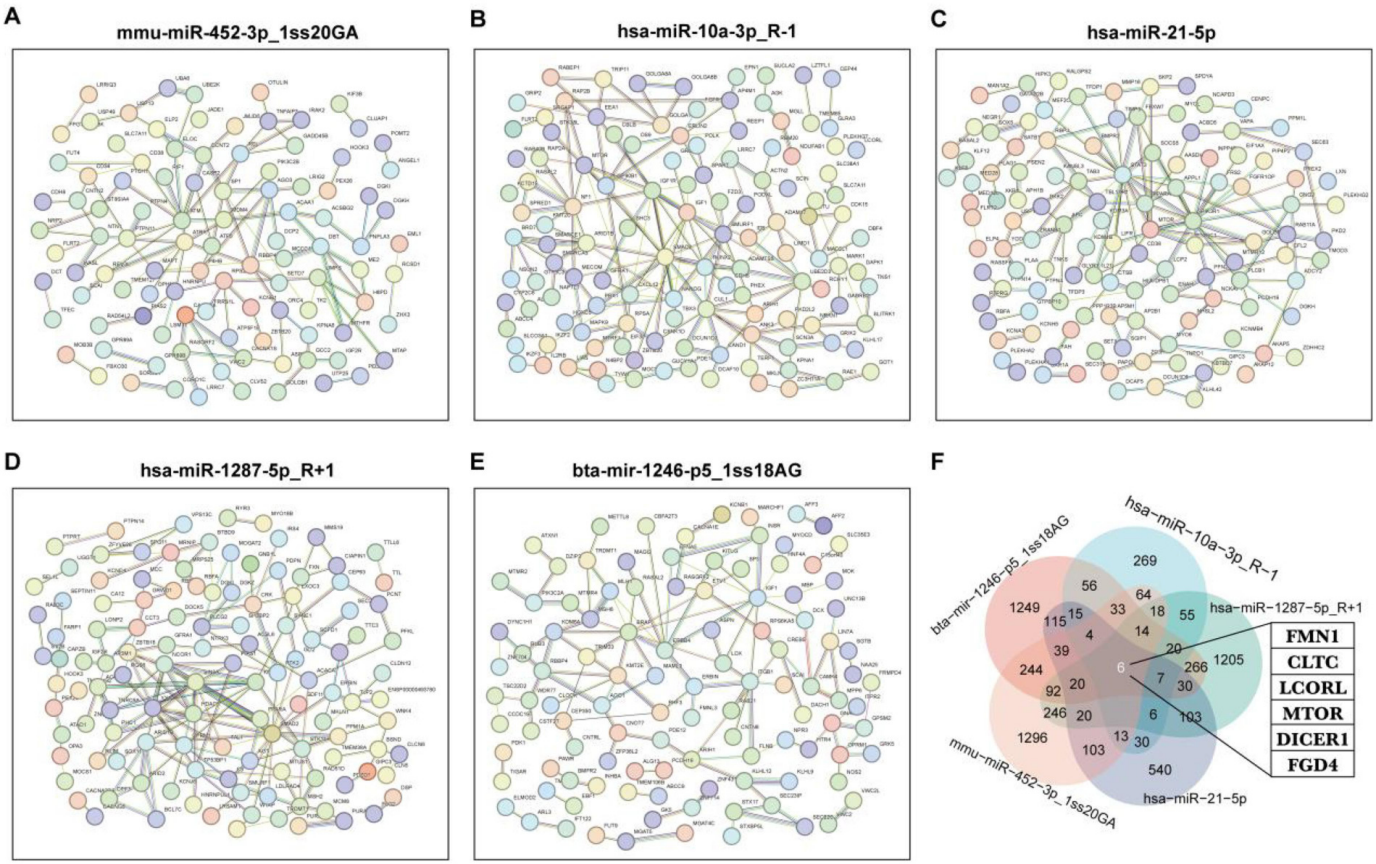
Interactions of target proteins of circulating miRNA biomarkers in patients with group 2 PH. **(A–E)** Protein interaction network of target proteins of target proteins of mmu-miR-452-3p_1ss20GA, hsa-miR-10a-3p_R-1, hsa-miR-21-5p, hsa-miR-1287-5p_R + 1 and bta-mir-1246-p5_1ss18AG (Top200). **(F)** Co-regulatory target genes of five circulating miRNA biomarkers.

The top 200 target genes were selected based on TargetScan score and Miranda for PPI analysis (Figures 5A–E). FMN1, CLTC, LCORL, MTOR, DICER1 and FGD4 are genes that are co-regulated by the five circulating miRNA biomarkers (Figure 5F).

## 4 Discussion

This study utilized high-throughput sequencing and experimental validation to identify a panel of circulating miRNAs that are significantly dysregulated in patients with Group 2 PH. Expression levels of five circulating miRNAs (mmu-miR-452-3p_1ss20GA, hsa-miR-10a-3p_R-1, hsa-miR-21-5p, hsa-miR-1287-5p_R + 1, and bta-mir-1246-p5_1ss18AG) were significantly correlated with key echocardiographic parameters (TRVmax and SPAP). These miRNAs exhibited high diagnostic accuracy, as reflected by their AUC values, suggesting their potential utility as non-invasive biomarkers for Group 2 PH. Furthermore, bioinformatic analyses indicated that these miRNAs participate in key biological processes and signaling pathways implicated in PH pathogenesis, including muscle cell differentiation, TGF-β signaling, JAK-STAT, MAPK, and p53 pathways.

The current diagnosis of PH relies heavily on right heart catheterization, which is invasive, costly, and not universally accessible (13). Echocardiography, though widely used, lacks the sensitivity and specificity for early detection or differential subtyping (5). There is thus an urgent need for readily measurable biomarkers to facilitate early diagnosis and reflect disease severity. Owing to their circulatory stability and pivotal functions in post-transcriptional regulation, miRNAs are considered promising candidates for biomarker development (14). Despite advances in characterizing tissue-specific miRNAs in PH (10, 15, 16), significant gaps persist in our understanding of circulating miRNAs, notably within the context of Group 2 PH. Our work helps fill this gap by identifying plasma miRNA signatures specific to PH complicating CHD.

Among the validated miRNAs, miR-21-5p has been extensively studied in cardiovascular contexts and is known to promote vascular smooth muscle proliferation and endothelial dysfunction (17). Its upregulation in our PH cohort aligns with its proposed role in vascular remodeling. Similarly, miR-10a-3p has been associated with cardiac hypertrophy and fibrosis, processes highly relevant to right ventricular adaptation in PH. The positive correlation between these miRNAs and pulmonary pressure measurements underscores their potential as indicators of hemodynamic severity.

The other miRNAs identified—such as mmu-miR-452-3p_1ss20GA and bta-mir-1246-p5_1ss18AG—are less well-characterized. Notably, bta-mir-1246-p5_1ss18AG, though annotated as a bovine miRNA, was detected in human plasma, consistent with previous reports of cross-species miRNA conservation or potential dietary origins (18). Its predicted involvement in RAS/MAPK signaling is of particular interest, given the central role of these pathways in pulmonary vascular cell proliferation (19).

Functional enrichment analysis revealed that the target genes of these miRNAs are involved in critical processes such as histone modification (mmu-miR-452-3p_1ss20GA), muscle differentiation (hsa-miR-10a-3p_R-1 and hsa-miR-21-5p), TGF-β signaling (hsa-miR-1287-5p_R + 1), and MAPK/RAP1 signaling (bta-mir-1246-p5_1ss18AG). These pathways are known contributors to pulmonary vascular remodeling and right ventricular dysfunction (20–22). Moreover, PPI analysis highlighted several hub genes—including FMN1, CLTC, LCORL, MTOR, DICER1, and FGD4—that are co-regulated by multiple candidate miRNAs. These genes have been implicated in cytoskeletal organization, MTOR signaling, and miRNA processing, suggesting a coordinated regulatory network influencing PH progression.

Several limitations should be acknowledged. First, First, the classification of pulmonary hypertension (PH) relied on clinical and echocardiographic criteria without mandatory confirmation by right heart catheterization, the diagnostic gold standard. While this reflects real-world diagnostic pathways, it may reduce the specificity of the Group 2 PH cohort and introduce phenotypic heterogeneity. Second, the sample size was relatively small, and all participants were male, limiting the generalizability of the findings. Future studies should include larger, gender-balanced cohorts and independent validation sets. Third, although bioinformatic predictions provide mechanistic insights, experimental validation using *in vitro* or *in vivo* models is necessary to confirm the functional roles of these miRNAs. Fourth, the study focused exclusively on Group 2 PH. Future comparisons with other PH subtypes will help evaluate the specificity of the identified biomarkers.

In conclusion, we identified a set of circulating miRNAs that are differentially expressed in Group 2 PH and correlated with disease severity. These miRNAs hold potential as novel diagnostic biomarkers and offer insights into disease mechanisms. Therefore, their therapeutic potential and clinical applicability merit further investigation.

## Data Availability

The datasets presented in this study can be found in online repositories. The names of the repository/repositories and accession number(s) can be found in the article/Supplementary Material.
